# Battling pain from osteoarthritis: causing novel cell death: Battling pain from osteoarthritis

**DOI:** 10.3724/abbs.2024189

**Published:** 2024-10-28

**Authors:** Yuheng Zhang, Huaqiang Tao, Liyuan Zhang, Xueyan Li, Yi Shi, Wen Sun, Wenlong Chen, Yuhu Zhao, Liangliang Wang, Xing Yang, Chengyong Gu

**Affiliations:** 1 Anesthesiology Department Suzhou Municipal Hospital (North District) Nanjing Medical University Affiliated Suzhou Hospital Suzhou 226000 China; 2 Department of Orthopedics the First Affiliated Hospital of Soochow University Suzhou 226000 China; 3 Orthopedics and Sports Medicine Center Suzhou Municipal Hospital Nanjing Medical University Affiliated Suzhou Hospital Suzhou 226000 China; 4 Department of Orthopedics the Affiliated Changzhou Second People’s Hospital of Nanjing Medical University Changzhou 213003 China

**Keywords:** osteoarthritis, pain, cell death, pyroptosis, ferroptosis, necroptosis

## Abstract

Osteoarthritis (OA) is a significant contributor to pain and disability worldwide. Pain is the main complaint of OA patients attending the clinic and has a large impact on their quality of life and economic standards. However, existing treatments for OA-related pain have not been shown to achieve good relief. The main focus is on preventing and slowing the progression of OA so that the problem of OA pain can be resolved. Pain caused by OA is complex, with the nature, location, duration, and intensity of pain changing as the disease progresses. Previous research has highlighted the role of various forms of cell death, such as apoptosis and necrosis, in the progression of pain in OA. Emerging studies have identified additional forms of novel cell death, such as pyroptosis, ferroptosis, and necroptosis that are linked to pain in OA. Different types of cell death contribute to tissue damage in OA by impacting inflammatory responses, reactive oxygen species (ROS) production, and calcium ion levels, ultimately leading to the development of pain. Evidence suggests that targeting novel types of cell death could help alleviate pain in OA patients. This review delves into the complex mechanisms of OA pain, explores the relationship between different modes of novel cell death and pain, and proposes novel cell death as a viable strategy for the treatment of these conditions, with the goal of providing scientific references for the development of future OA pain treatments and drugs.

## Introduction

The prevalence of osteoarthritis (OA) may increase due to increasing average life expectancy and the world’s population. OA is often accompanied by pain, which negatively affects a patient’s physical function, quality of life and economic status. OA is a prevalent long-term condition affecting joints and is characterized by pain, swelling and restricted movement. OA damage is extensive and involves various parts of the joint. Painful joint alterations, such as synovitis and myelopathy, are related to abnormal cytokine expression or increased sensitivity to pain stimuli [ 1 , 2]. Pain is the most prevalent symptom and leading cause of disability in OA patients. Similarly, OA-related pain involves various inflammatory cytokines, structural joint changes, and neuropathic pain [3]. OA pain relief involves multiple mechanisms, and targeting inflammation and neuroinflammation; modulating specific signaling pathways; and inhibiting pyroptosis, ferroptosis, and necroptosis may be effective ways to reduce OA pain. Inflammation is an important factor in the development of OA pain, and neuroinflammation, in particular, plays a significant role in the development of OA pain. The development of pyroptosis, ferroptosis and necroptosis is associated with inflammatory responses, and inflammatory cytokines are a common cause of pain. 

Notably, the development of pain caused by OA is similar to that caused by other types of pain. As a result, several medications, such as non-steroidal anti-inflammatory drugs and opioids, have been developed for treating pain. However, these drugs cannot provide adequate pain relief and have severe side effects, such as gastrointestinal harm, addiction, and hindrance in bone healing and remodeling [ 4‒ 6]. Although surgery can be used to treat OA, it can also cause more pain in the short term than the original disease can and even increase the risk of postoperative complications and chronic pain. Therefore, drugs with better efficacy and fewer side effects are needed to relieve pain and slow disease progression [ 7, 8]. 

Multiple studies have shown that novel types of cell death are significantly related to OA [ 9‒ 13]. Therefore, further studies should elucidate the mechanisms by which various types of cell death affect disease development. Research has demonstrated that various pharmacological or non-pharmacological treatments can alleviate OA-related pain by modulating cell death. For example, electroacupuncture and ibuprofen can alleviate the pain symptoms of primary dysmenorrhea by inhibiting the expression of NOD-like receptor thermal protein domain associated protein 3 (NLRP3) and thereby reducing cellular pyroptosis [14]. Components such as mangiferin, cinnamic acid, and vanillic acid showed potential therapeutic effects in modulating inflammation and relieving pain, particularly in inhibiting NLRP3 inflammatory vesicle activation and combating oxidative stress [ 15, 16]. Pharmacologic treatment of OA pain has focused on symptomatic relief, including the use of NSAIDs, COX-2 inhibitors, and antidepressants. Deferoxamine and edaravone (iron chelators and free radical scavengers, respectively) prevent cellular ferroptosis and alleviate the pain associated with experimental autoimmune prostatitis [17]. For example, ferrostatin-1, an inhibitor of ferroptosis, inhibits ERK1/2 and COX-2 expression and attenuates pain-associated activation [18]. Lipoxin A4 inhibits ferroptosis in FLSs through the activation of the ESR2/LPAR3/Nrf2 axis, which attenuates pain and pathological progression in OA [19]. Melatonin may exert analgesic effects on chronic pain by inhibiting necroptosis [20]. Necrostatin-1, an inhibitor of RIP1, is able to protect rats from trauma-induced cartilage degradation and limb pain by inhibiting RIP1 activity [21]. Green-lipped mussels also reduced the expression levels of the necroptosis mediators RIPK1, RIPK3, and mixed-spectrum kinase structural domain-like protein (MLKL), which are important molecules in the necroptosis pathway that improve pain level by preventing cartilage damage and inflammation, in IL-1β-stimulated human OA chondrocytes [22]. 

This review aimed to examine how pain occurs in OA and the impact of different types of cell death on pain. The impact of therapeutic agents on cell death was also evaluated to guide the development of new clinical treatments.

## General Treatment of OA

The major existing international treatment guidelines recommend non-pharmacological treatments for OA, such as patient education, exercise therapy and weight loss [23]. Moreover, the Osteoarthritis Research Society International, American College of Rheumatology, and European Society for Clinical and Economic Aspects of Osteoporosis and Osteoarthritis guidelines all recommend topical non-steroidal anti-inflammatory drugs as the first-line treatment owing to their definite efficacy on the painful symptoms of OA and the lower risk of gastrointestinal, cardiovascular, and renal adverse effects. Although surgery should be used as a treatment for end-stage OA, it is associated with more complications, including venous thrombosis, infection, pulmonary embolism, and death [ 24‒ 26]. 

Nonetheless, these treatments only relieve symptoms, such as pain, and may produce certain side effects. As a result, effective treatments or therapeutic targets are necessary. As the understanding of the pain and pathophysiologic mechanisms of OA has deepened and as treatments targeting both peripheral- and central-related pathologic processes have failed to provide good and sustained clinical outcomes, researchers have begun to consider whether there are other more effective treatments or therapeutic targets.

## Pain Mechanisms in OA

Pain is a disagreeable sensory and emotional feeling linked to real or possible harm to tissues, according to the International Association for the Study of Pain [27]. Pain in OA occurs when a stimulus to the bone directly excites or sensitizes sensory fibers on the periosteum, bone cortex, and bone marrow, inducing the ectopic growth of sensory fibers through the upregulation of inflammatory mediators, receptors, and ion channels. This over-innervates the bone and centrally sensitizes the bone to amplify the pain. Notably, a normal innocuous stimulus can be perceived as noxious and can lead to chronic pain. The dorsal root ganglion serves as the main sensory structure in the vertebrate nervous system, sending peripheral sensory signals to the central nervous system. Increased activity in sensory neurons near the dorsal root ganglion indicates heightened pain sensitivity caused by injury or illness [ 28‒ 30]. The production of local inflammatory pain is associated with the activation or sensitization of injurious afferent nerve fibers caused by pro-inflammatory cytokine-mediated inflammation [31]. These fibers carry pain signals to the dorsal horn of the spinal cord, which travel through various pathways to nociceptive centers in the cerebral cortex, where pain is experienced. Pro-inflammatory cytokines can also directly activate injury receptors, as well as neuroglia and immune cells, thereby causing pain [ 32, 33]. Cartilage degradation products produced during joint injury may directly cause pain by directly stimulating injured neurons and glial cell activation [34]. An increased number of microglia within the central nervous system significantly leads to the development of chronic pain conditions, such as neuropathic pain and inflammatory pain [35]. 

### Biological mechanism

Pain in OA is usually associated with a number of factors, including wear and tear of the cartilage, inflammation of the synovium, bone marrow lesions, and changes in the structures surrounding the joint [36]. The cartilage itself is not innervated and therefore does not directly cause pain. However, in the early stages of OA, cartilage fragments may be engulfed by cells in the synovium, triggering an inflammatory response and synovitis, which can be a source of pain [37]. As the disease progresses, the mechanical supportive role of subchondral bone, metabolic regulation, and innervation become more important [38]. Subchondral vascularization and bone remodeling may be associated with pain, as they may promote nerve growth and innervation of sensory nerves. In more severe OA, the onset of pain is closely related to neurovascular invasion at the osteochondral junction. Osteoclasts play a key role in subchondral bone remodeling, and their secretion of NETRIN1 may induce sensory innervation and pain via the receptor deleted in colorectal cancer (DCC) [39]. Drugs such as alendronate can inhibit osteoclast activity, thereby reducing innervation and pain behavior in the early stages of OA. In addition, molecules such as parathyroid hormone and miR-204 have been shown to reduce OA pain [ 40, 41]. 

Synovitis is another important factor in OA pain, especially in mild to moderate OA. Increased synovitis scores are associated with increased knee pain, and synovitis may be related to the rate of disease progression [42]. Synovitis can exacerbate cartilage damage, and abnormal proliferation of blood vessels in the synovium is also strongly associated with pain [37]. Urolithin A attenuates disease progression and reduces cartilage degeneration, synovial inflammation and pain in a mouse model of OA [43]. Exosomes derived from bone marrow-derived mesenchymal stem cells can effectively promote cartilage repair and extracellular matrix synthesis, alleviate knee pain, reduce CGRP and iNOS protein levels, and alleviate the protective effects of inflammatory and neuropathic pain in OA rats [44]. Bone marrow lesions are another potential cause of OA pain, especially in areas with high loads. Bone marrow lesions are characterized by fat necrosis, localized myelofibrosis, and trabecular microfractures, all of which are associated with active bone remodeling and repair. Interventions to reduce knee OA loading may help minimize myelopathy and knee pain [45]. Table 1 focuses on OA pathologic features that cause pain.

**Table:** **Table 1** Pathologic features of pain due to OA

Tissue/cell	Pathological manifestations causing pain	Key signaling pathway	Ref.
Synovium	Synovial lining hyperplasia, increased vascularization, and inflammatory cell infiltration	MMP3/MMP13/MMP9, TNF/ADAMTS­4	[ 2, 10, 37]
Subchondral bone	Bone remodeling of subchondral bone, bone marrow edema and generation of osteoclasts, H-type angiogenesis	OPG/RANKL/RANK, Wnt signaling pathway, TGF-β1-Smad2/3, RANKL-VEGF-ERK1/2	[ 46, 47]
Infrapatellar fat pad	Increased vascularization, inflammatory infiltration	integrin β1/ERK/VCL, OPN/integrin β3	[ 48, 49]
Meniscus and anterior cruciate ligament	Mechanical damage, neovascularisation and innervation	Wnt signaling pathway, CLK2/DYRK1A	[ 50‒ 52]

Pain in OA may be felt as pins and needles or burning, paroxysmal or constant, and can occur after activity or at rest, and is a mixture of peripheral and central pain. Pain also causes physical and psychological dysfunction secondary to decreased mobility, bone loss, loss of muscle mass, and decreased cognitive and cardiovascular health, all of which reduce quality of life. Pain is significantly associated with functional limitations, and risk factors that exacerbate pain are prevalent in contemporary lifestyle habits, such as reduced activity, a sedentary lifestyle, and obesity [ 53, 54]. These factors reduce bone and muscle health while accelerating the progression of OA. 

### Molecular mechanism

However, research has focused only on the pathophysiology of pain. Multiple pain-related molecular signaling pathways participate in pain pathophysiology. Numerous pathways, including the MAPK pathway, the Wnt pathway, the Janus kinase (JAK)/signal transducer and activator of transcription (STAT) signaling pathway, the NF-κB pathway, the NGF/pro-myosin receptor kinase (TrkA) signaling pathway, the NO/cGMP signaling pathway, and the extracellular signal-regulated kinase (ERK)/cAMP-response element binding protein pathway, are crucial in the progression and persistence of OA, particularly chronic pain [ 55, 56]. Increased expressions of pain-related genes such as *PGE2*, *CGRP*, and *TRPV1* cause direct pain, and pain relief can be achieved by modulating the upstream signaling pathways of these genes [57]. 

The MAPK family includes ERK, p38 and c-Jun N-terminal kinase (JNK) [58]. These pathways can be activated by a variety of inflammatory factors and play key roles in the regulation of chronic nociceptive sensitization. The activation of JNK is associated with the production of pro-inflammatory cytokines (TNF-α, IL-1, and IL-6), cellular stress, and reactive oxygen species (ROS), which can trigger the progression and persistence of pain [59]. The Wnt pathway is closely associated with pain, and its activation increases the release of inflammatory molecules and exacerbates the development of neuropathic pain. The Wnt/β-catenin signaling pathway plays a role in controlling the development of arthritis while maintaining chondrocyte viability, and inhibition of the Wnt signaling pathway reduces the expression of the pain-related molecules iNOS and COX-2 [60]. Different stimuli activate different JAK/STAT pathways in synovial macrophages from patients with OA, which mediate macrophage polarization toward the M1 or M2 phenotype, resulting in proinflammatory or anti-inflammatory effects, and it has been demonstrated that inhibition of JAK2/STAT3 contributes to a reduction in COX-2 expression [ 61 ‒ 63]. JAK inhibitors may modulate pain directly or indirectly by inhibiting this pathway. The NF-κB pathway regulates inflammatory and neuropathic pain through inflammatory cytokines such as IL-6, TNF, and IL-1β. Activation of the NF-κB signaling pathway promotes the apoptosis of chondrocytes in OA, which leads to pain and inflammation [ 59, 64]. The inhibition of NF-κB reduces the expression levels of COX-2 and iNOS, resulting in pain relief [65]. The NGF/TrkA axis may be involved in synovial fluid homeostasis and increased pain sensitivity in patients with knee OA. NGF is itself a peripheral pain mediator, and NGF-induced pain may be associated with increased nerve growth, irregularities at the bone-cartilage junction, and peripheral sensitization [ 66, 67]. The activation of NO/cGMP signaling in the spinal cord plays a role in astrocyte proliferation and microglial polarization in chronic pain [68]. Analgesic effects are dependent on activation of the sGC/cGMP/protein kinase G pathway [69]. 

The GM-CSF/CCL17 pathway has been recognized as a key factor when the mechanisms of OA pain are explored. This pathway promotes neuronal sensitization through CCR4-expressing cells and affects obesity-related pain symptoms [70]. In OA pain, GM-CSF triggers inflammation via the GM-CSF/Jmjd3/IRF4/CCL17 pathway, and the inhibition of CCL17 ameliorates GM-CSF-induced inflammatory pain, arthritic pain, and diseases [71]. In addition, angiogenesis in cartilage is also associated with pain, and the expression of Yes-associated protein 1 (YAP1) and piezoelectric-type mechanosensitive ion channel component (Piezo)1 receptors causes pain through vascular remodeling [ 72, 73]. Piezo2 is associated with mechanotransduction of somatosensory sensations such as touch, proprioception, and pain, and activation of exchange proteins by cAMP1 is critical in inflammatory pain [48]. The Hippo/YAP1 signaling pathway is involved in the cellular stress response, which promotes osteophyte formation and limits joint motion, thereby exacerbating pain [ 74, 75]. In cases of persistent inflammatory pain induced by complete Freund’s adjuvant, T-type Cav3.2 channels promote pain transmission through the IGF-1/IGF-1 receptor pathway, and IGF-1 can also exacerbate pain by increasing TRPV1 currents [76]. Glial-cell line-derived neurotrophic factor (GDNF) family ligands, including GDNF, neurturin, and artemin, act through the GDNF family receptor (GFRα) to induce inflammatory bone pain. The artemisinin/GFRα3 signaling pathway activates and sensitizes peptidergic NGF-sensitive neurons, and the GDNF/GFRα1 and neurturin/GFRα2 signaling pathways activate and sensitize nonpeptidergic neurons; thus, chelation of artemisinin or neurturin can be used to treat inflammatory bone pain [77]. These signaling pathways influence the expressions of pain-related genes, and by regulating the expression of these signaling pathways, effective analgesia may be obtained. 

## Targeting Cell Death Can Treat Pain in OA

Cell death marks the end of cell life and is the final development of cells in various pathophysiological situations. Studies have demonstrated that chondroitin sulfate from sturgeon bone [77], malvidin [64], and the ancient Chinese remedy Agkistrodon [78] can alleviate pain in OA rats by preventing osteoclast apoptosis. Hydrogen can attenuate tert-butyl hydroperoxide (TBHP)-induced apoptosis and extracellular matrix degradation in human chondrocytes by inhibiting the JNK signaling pathway [79]. 

Pyroptosis is a type of caspase-mediated cell death. The biochemical and morphological characteristics of cells undergoing pyroptosis include apoptotic and necrotic cells. Exposure to pathogens (bacteria or viruses) or substances derived from pathogens can initiate the formation of the inflammatory vesicle NLRP3 and the activation of caspase-1 [80]. Active caspase-1 cleaves pro-IL-1β and pro-IL-18 and can trigger gasdermin D (GSDMD) cleavage, revealing the N-terminal structural domain, thus allowing pore formation on the plasma membrane [81] .

Ferroptosis, a type of cell death that relies on iron and lipid peroxidation (MDA), is caused mainly by iron buildup, ROS generation, and polyunsaturated fatty acid peroxidation in phospholipids, leading to the release of pro-inflammatory substances. Unlike other forms of cell death, ferroptosis does not induce nuclear morphological changes, DNA breaks, or caspase-3 activation.

Necroptosis does not depend on cysteine asparaginase but rather on an internal signaling pathway through receptor-interacting protein kinase (RIPK) 3. Necroptosis involves a combination of apoptosis and necrosis, increasing the levels of cytoplasmic calcium ions and ROS, reducing ATP, and causing internal acidification. This results in cell swelling and the release of damage-associated molecular patterns (DAMPs) when the plasma membrane ruptures. TNF-α and TNFR1 activate the receptor-interacting protein 1/RIP3/MLKL necrosome independently of caspases, leading to cell death through membrane swelling and plasma membrane rupture, resulting in the release of DAMPs [82] . Table 2 focuses on the cellular morphological features and the key signaling pathways induced by various modes of cell death.

**Table:** **Table 2** Morphological features of cells induced by various modes of cell death and key signaling pathways mediating death

Cell death	Cell morphological characterization	Key pathway axis	Ref.
Apoptosis	Shrunken cell, intact cell membrane, lost mitochondrial membrane potential, condensed nucleoplasm, and fragmented nuclear membrane and nucleolus.	Executed by caspase-3/7	[11]
Pyroptosis	Cell swelling, perforation of the plasma membrane, and release of cell contents.	NLRP3/caspase-1/GSDMD	[ 81, 83]
Ferroptosis	Cell swelling, rupture of the plasma membrane, increased density of the mitochondrial membrane, rupture of the outer membrane, reduction or disappearance of the mitochondrial cristae	Caused by iron overload resulting in MDA	[84]
Necroptosis	Cell swelling, plasma membrane rupture, organelle swelling, mild chromatin condensation	RIPK1-RIPK3-MLKL	[85]

### Fibroblast-like synoviocytes

Significantly increased DAMP levels in OA synovial fluid trigger typical inflammasome pathway-mediated pyroptosis in chondrocytes, fibroblast-like synoviocytes (FLSs), or synovial macrophages (SMs), causing pathological changes in the synovial membrane and promoting OA pain production. DAMPs mediate the subsequent pyroptosis of the synovium by interacting with SMs and the membrane TLRs of FLSs.

Increased levels of miR-20a suppress FLS pyroptosis in an adjuvant-induced arthritis model by reducing TXNIP expression, thus decreasing the levels of NLRP3, apoptosis-associated speck-like protein (ASC), caspase-1, and IL-1β. This effect eases pain symptoms related to pyroptosis and delays the progression of arthritis [13]. Secreted frizzled-related protein 1 (SFRP1) controls the growth, development, and transformation of cells. SFRP1 acts as an antagonist of the Wnt signaling pathway, disrupting Wnt signaling and influencing cell proliferation, differentiation, apoptosis, and pyroptosis. The levels of proteins, such as Wnt3a, Wnt5a, and Wnt10a, increase in FLSs, triggering the Wnt signaling pathway and downstream genes. This elevates fibronectin expression; enhances cell proliferation, migration, and survival; and stimulates the growth of synovial tissues even in the absence of pro-inflammatory factors [86]. 

The activation of the Notch1 pathway and control of its target genes impact several functions, including cell growth and movement in FLS, while significantly increasing the expression of Notch3 and its target genes in FLS. SFRP1 can attach to ADAM metallopeptidase domain 10 within the Notch signaling pathway in patients, decreasing its function and preventing the activation of the Notch signaling pathway. Therefore, SFRP1 may participate in NLRP3-induced pyroptosis through the modulation of the Wnt/β-catenin and Notch pathways in FLSs [86]. 

**Figure 1:**
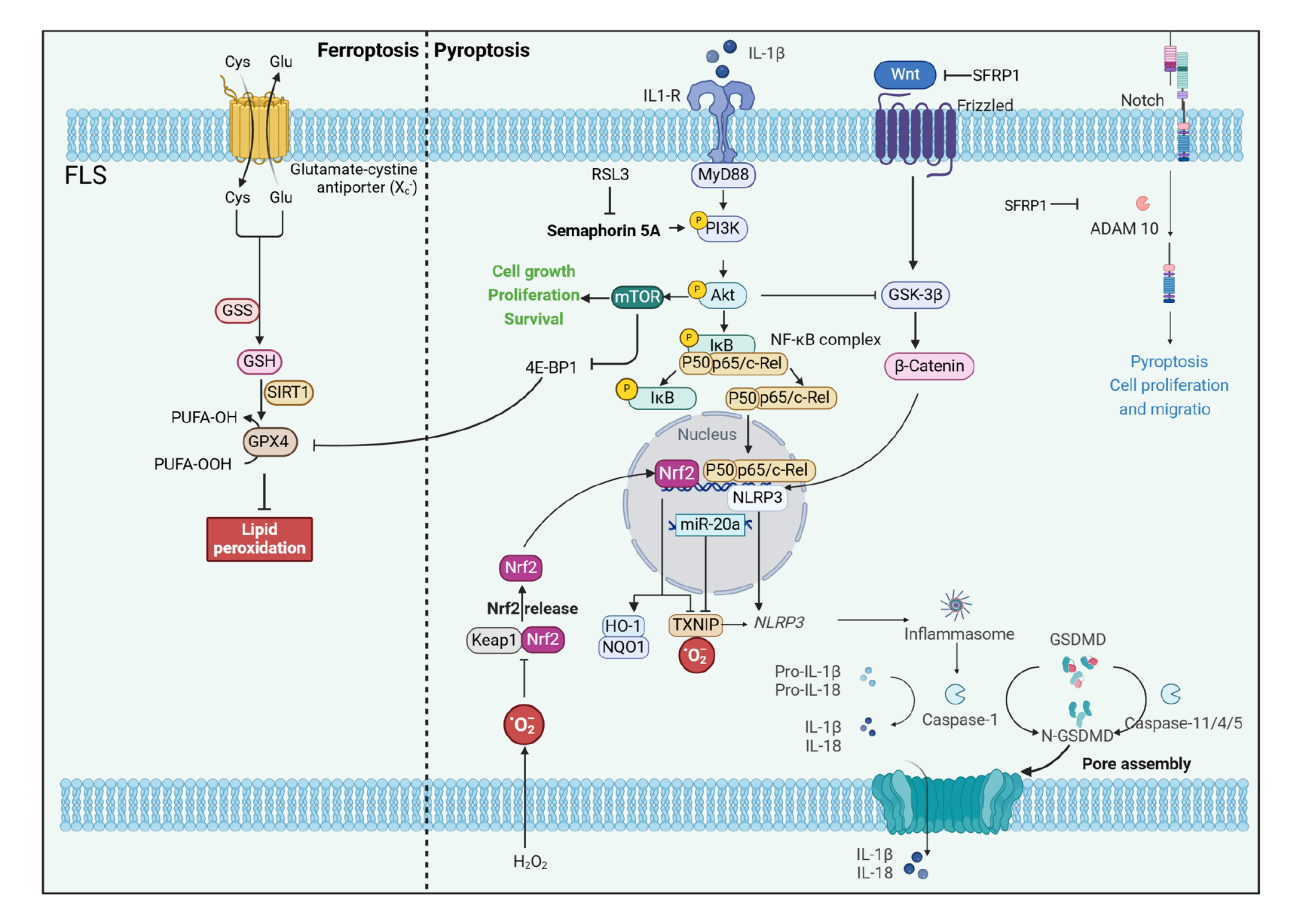
Figure 1 The metabolic pathways of FLS pyroptosis and ferroptosis are involved in the onset of pain in OA and the interplay between response components and those involved in other modes of cell death The left half shows the FLS pyroptosis-related signaling pathways. Many factors trigger, through the same or different pathways, the assembly of NLRP3 inflammatory vesicles and the subsequent activation of caspase-1 or caspase-11/4/5, which cleaves GSDMD proteins and pro-IL-1β and pro-IL-18, leading to the release of IL-1β and IL-18 and the release of the cell through the channels formed by N-GSDMD. ROS upregulates TXNIP expression through the inhibition of Nrf2/HO-1, further accelerating the onset of pyroptosis. miR-20a overexpression inhibits FLS pyroptosis through the downregulation of TXNIP expression. SFRP1 is a Wnt signaling pathway antagonist. SFRP1 can bind to and downregulate the activity of the ADAM metallopeptidase domain 10 metalloprotein of the Notch signaling pathway, thereby blocking the activation of the Notch signaling pathway. SFRP1 may be involved in NLRP3-mediated pyroptosis by regulating the Wnt/β-catenin and Notch signaling pathways. The right half of the figure shows the FLS ferroptosis-related signaling pathway. Semaphorin 5A significantly upregulates GPX4 expression by activating the PI3K/Akt/mTOR signaling pathway, which is attenuated by RSL3-induced ferroptosis. Activation of the PI3K/Akt/mTOR signaling pathway also promotes GPX4 protein translation by inhibiting the downstream molecule 4E-BP1. Knockdown of SREBP1 and SCD-1 significantly abrogates the reduction in MDA levels caused by semaphorin 5A, while it has no effect on ROS levels.

The expression of GPX4 is decreased during cellular ferroptosis. Semaphorin 5A strongly increases the RNA and protein expressions of GPX4 in FLSs by activating the PI3K/AKT/mTOR pathway. However, RSL3-induced ferroptosis can reduce the RNA and protein expressions of GPX4. In addition, activation of the PI3K/AKT/mTOR pathway enhances GPX4 protein synthesis by suppressing 4E-BP1, thus preventing cellular ferroptosis. Co-treatment of cells with semaphorin 5A and RSL3 significantly decreases MDA and ROS levels and improves mitochondrial morphology [ 87, 88]. Although knockdown of *SREBP1* and stearoyl coenzyme A desaturase-1 ( *SCD-1*) can reverse the effect on MDA, it does not affect ROS levels. Therefore, the SREBP1/SCD-1 signaling is crucial in ferroptosis, suggesting that it is a potential target for suppressing cellular ferroptosis and offering a novel approach for managing arthritis-related pain [87] ( Figure 1).

### Synovial macrophages

High mobility protein B1 interacts with FLSs to cause the accumulation of inflammatory molecules that result in synovitis [ 73, 89]. Additionally, high mobility protein B1 binds to LPS, which is then transported to SMs through RNA, inducing pyroptosis [90]. Furthermore, TNF-α is generated and secreted primarily by monocytes and nearby macrophages, which are activated by TNF-α to produce additional TNF-α in an OA model, leading to a feedback loop that enhances pyroptosis initiation. TNF triggers monocyte/macrophage pyroptosis in patients by inducing the activation of caspase-3/GSDME signaling. Studies have shown that patients with elevated GSDME levels in their peripheral blood monocytes are more likely to experience pyroptosis and are at increased risk of spontaneous GSDME-induced pyroptosis [91]. 

**Figure 2:**
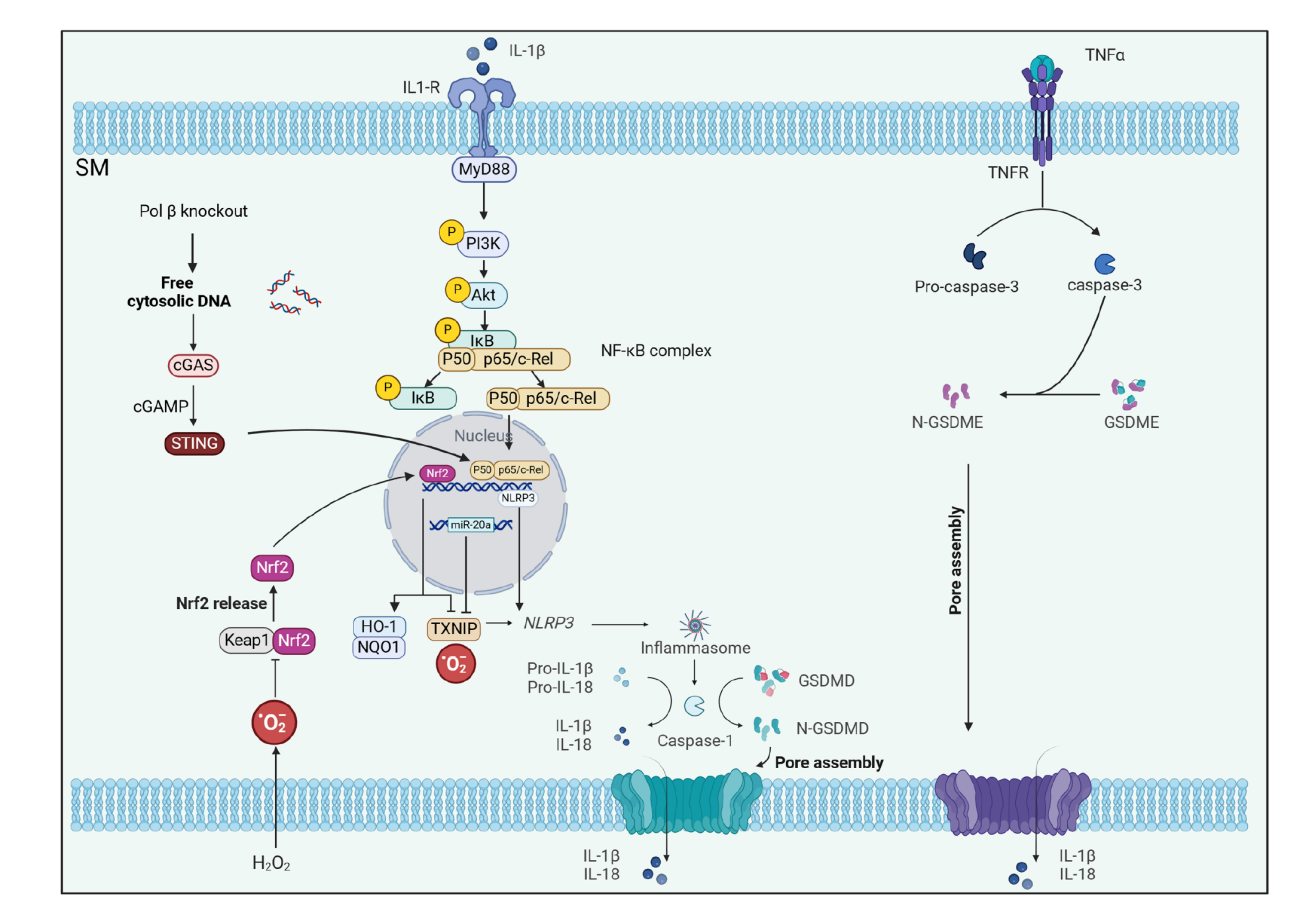
Figure 2 Metabolic pathways involved in SM pyroptosis during OA pain onset and the interplay between the reactive components and those involved in other modes of cell death HMGB1 binds to LPS and is transported to SMs via RNA, leading to SM pyroptosis. TNF stimulates further production of TNF by monocytes and SMs, which in turn creates positive feedback and promotes pyroptosis. Positive feedback. TNF-α triggers SM death by inducing the activation of caspase-3/GSDME signaling. Downregulation of DNA polymerase β leads to the accumulation of DNA damage and the leakage of cytoplasmic dsDNA, which activates the cyclic GMP-AMP synthase/STING/NF-κB signaling pathway and increases the expressions of NLRP3, IL-1β, and IL-18. HMGB1: high mobility protein B1.

Additionally, the inhibition of SM pyroptosis induced by GSDMD siRNA significantly downregulated synovial fibrosis-related indices in the FLSs of OA patients. Therefore, the inhibition of SM pyroptosis may reduce the degree of fibrosis in OA joints [92]. Baihu-Guizhi decoction therapy decreases redness in joints and increases the level of pain in adjuvant-induced arthritis rats. Baihu-Guizhi decoction therapy can also decrease the levels of TLR4 proteins and proteins related to pyroptosis, such as NLRP3, GSDMD, and caspase-1 [93]. These findings demonstrate that LPS/ATP may promote SM pyroptosis through TLR4-mediated activation of NLRP3 signaling [93]. Previous studies have shown that reducing DNA polymerase β in OA mice increases DNA damage and leakage of cytoplasmic dsDNA. This triggers the cyclic GMP-AMP synthase/STING/NF-κB signaling pathway, increasing the expressions of NLRP3, IL-1β, and IL-18, and worsening the degree of macrophage pyroptosis induced by LPS and ATP [94] ( Figure 2 ).

### Chondrocytes

The phosphorylation of I-κB is increased in OA, thus promoting the translocation of p65 into the nucleus and activating NF-κB signaling and cellular pyroptosis. p65 is also an upstream activator of chondrocyte dysfunction and pyroptosis. The *NLRP3* gene also encodes another key component of the inflammatory vesicle, cryopyrin. Cryopyrin binds to the structural domain of the cysteine asparaginase recruitment domain of ASC and induces pro-caspase-1 recruitment. The expressions of two key proteins, cryopyrin and caspase-1, were increased in chondrocytes after OA modelling. It has been reported that loganin from the ancient Chinese remedy Corni Fructus can decelerate cartilage breakdown, prevent chondrocyte pyroptosis, and decrease abnormal blood vessel formation by blocking the NF-κB pathway and suppressing the production of pyroptosis-related proteins. As a result, Corni Fructus can protect cartilage and relieve the painful symptoms of OA [95]. 

**Figure 3:**
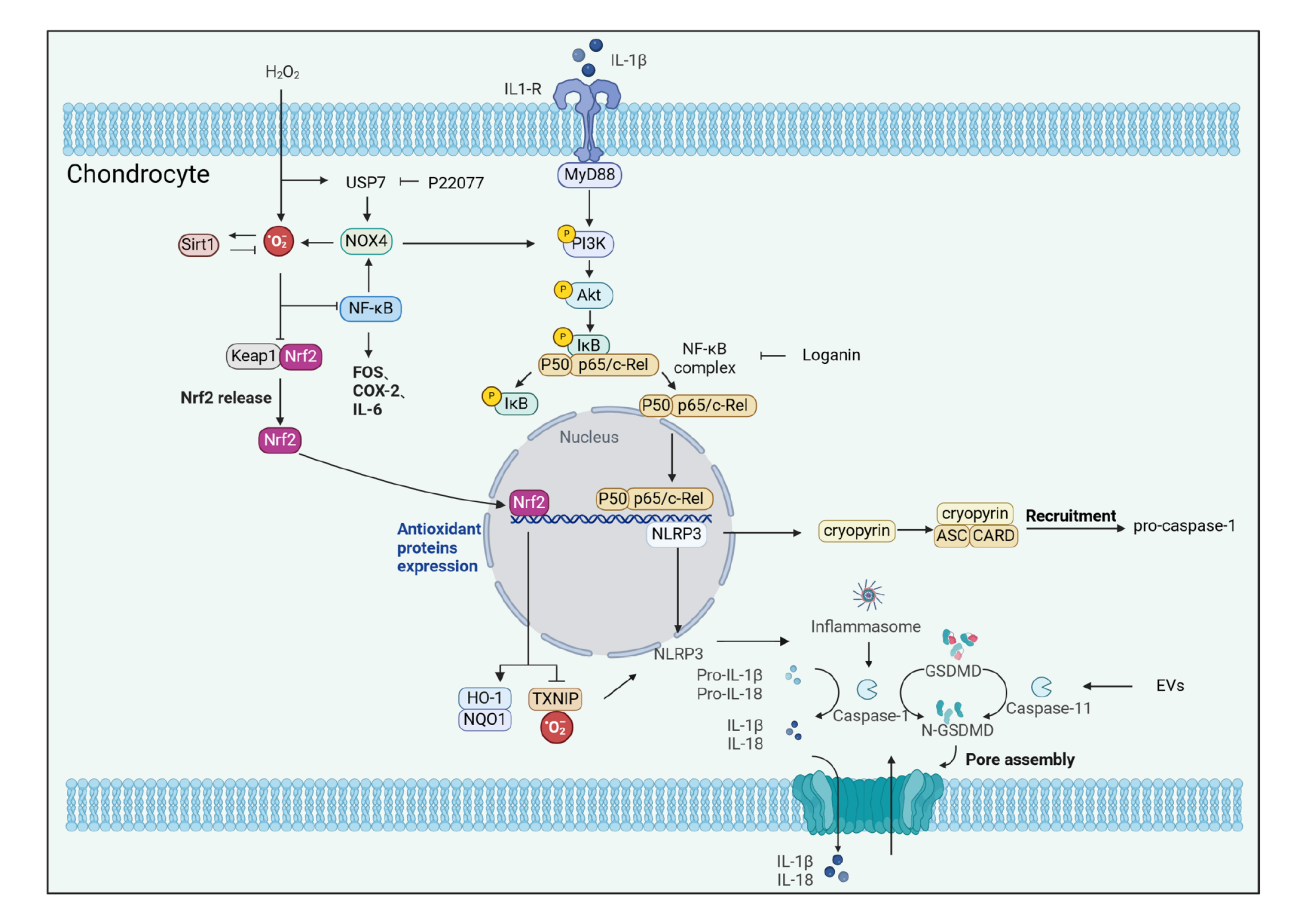
Figure 3 Chondrocyte pyroptosis pathways involved in pain onset in OA Many factors trigger, through the same or different pathways, the assembly of NLRP3 inflammatory vesicles and the subsequent activation of caspase-1 or caspase-11/4/5, which cleaves GSDMD proteins and pro-IL-1β and pro-IL-18, leading to the release of IL-1β and IL-18 from cells through the N-GSDMD-forming channels that are released from the cell. Inflammatory macrophage-derived exosomes promote chondrocyte pyroptosis by activating caspase-11/GSDMD signaling in chondrocytes. The NLRP3 gene encodes cryopyrin, an essential component of the inflammatory vesicle, which binds to the cysteine asparaginase recruitment domain structural domain of ASC and induces pro-caspase-1 recruitment. IL-1β binds to IL-1R through the PI3K/Akt pathway, triggering the assembly of NLRP3 inflammatory vesicles. ROS further accelerate pyroptosis through the inhibition of Nrf2/HO-1. H2O2 induces USP7-dependent deubiquitylation of NLRP3 through the induction of USP7-dependent deubiquitylation of NLRP3 and the activation of NOX4/HO-1, a DNA-binding component of NLRP3 that is able to bind to DNA. DNA-binding NOX4/ROS/NF-κB signaling increases the protein levels of NLRP3 and pro-caspase-1. The USP7 inhibitor P22077 attenuates H2O2-induced damage to chondrocytes and death through the inhibition of the NOX4/NLRP3 signaling pathway.

Studies have also shown that exosomes from inflammatory macrophages can induce chondrocyte pyroptosis in OA and anterior cruciate ligament transection OA models by activating caspase-11/GSDMD signaling in chondrocytes. Inhibition of caspase-11 greatly reduces pyroptosis and breakdown processes in activated chondrocytes, the damage caused by collagenase, and joint instability in an OA model [96]. The USP7 inhibitor P22077 reduces H _2_O _2_-induced damage to chondrocytes and pyroptosis by blocking the NOX4/NLRP3 signaling pathway [97] ( Figure 3).

NF-κB pathway activation may prevent ferroptosis in chondrocytes by promoting the production of hypoxia-inducible factor 2α, hindering GSH-GPX4 [98]. Ruscogenin suppresses MDA, iron buildup, and ROS generation while increasing the levels of GSH, GPX4, ferritin, Nrf2, and SLC7A11, thus preventing IL-1β-induced ferroptosis [99]. Furthermore, *Nrf2* knockdown reversed the suppressive effects of Rus on IL-1β-triggered inflammation, MMP generation, and ferroptosis. Kukoamine can reverse IL-1β-induced negative effects related to ferroptosis by increasing the levels of GSH, GPX4, ferritin, SIRT1, Nrf2, and HO-1. Furthermore, a SIRT1 inhibitor can reverse the suppressive effects of kukoamine on IL-1β-triggered inflammation, MMP synthesis, and ferroptosis [100]. Taken together, these findings suggest that IL-1β promotes chondrocyte ferroptosis through the Nrf2/SLC7A11/GPX4 and SIRT1/GPX4 signaling pathways [101]. Similarly, brevilin A can inhibit inflammatory responses and ferroptosis by modulating the SIRT1/Nrf2/GPX4 signaling [102]. 

MYC transcriptionally inhibits the *ADORA2B* gene, thus promoting ferroptosis in chondrocytes by blocking the PI3K/Akt pathway. Increased ADORA2B levels enhance ferroptosis. LY294002, an inhibitor of the PI3K/Akt pathway, can reverse the ferroptosis-promoting effect of ADORA2B. MYC functions as a transcriptional inhibitor of ADORA2B, increasing the survival of chondrocytes and decreasing susceptibility to ferroptosis. Conversely, ADORA2B upregulation counteracts the prosurvival impact of MYC on chondrocytes [103]. 

Transient receptor potential ankyrin 1 (TRPA1), a pain-associated indicator, contributes to pain signal propagation and has been detected in various sensory neurons and non-neuronal cells [ 104, 105]. In an OA model, the expression levels of TRPA1 were significantly increased in both wild-type and AMPKα-KO mice. Baicalein treatment increased the expression level and activation of AMPKα and suppressed the level of TRPA1 in the dorsal root ganglion but did not affect the levels of TRPA1, Nrf2, or HO-1 in the AMPKα-KO mice. The administration of ferrostatin-1, a ferroptosis inhibitor, decreased the OA surgery-induced increase in normal mice, thereby alleviating pain sensitivity. Baicalein has also been found to suppress chondrocyte ferroptosis and alleviate OA pain by activating the AMPKα/Nrf2/HO-1 signaling pathway [106] .

A recent study demonstrated that excessive mechanical stress induces ferroptosis in OA chondrocytes by activating Piezo1 and increasing intracellular Ca ^2+^ level. SLC3A2 is another subunit of System Xc ^–^, which is known to promote or delay the progression of OA by regulating chondrocyte ferroptosis. SLC3A2 downregulation promoted OA-associated chondrogenic degeneration, whereas ferrostatin-1 abolished the effects of *SLC3A2* knockdown on chondrocytes [107]. 

**Figure 4:**
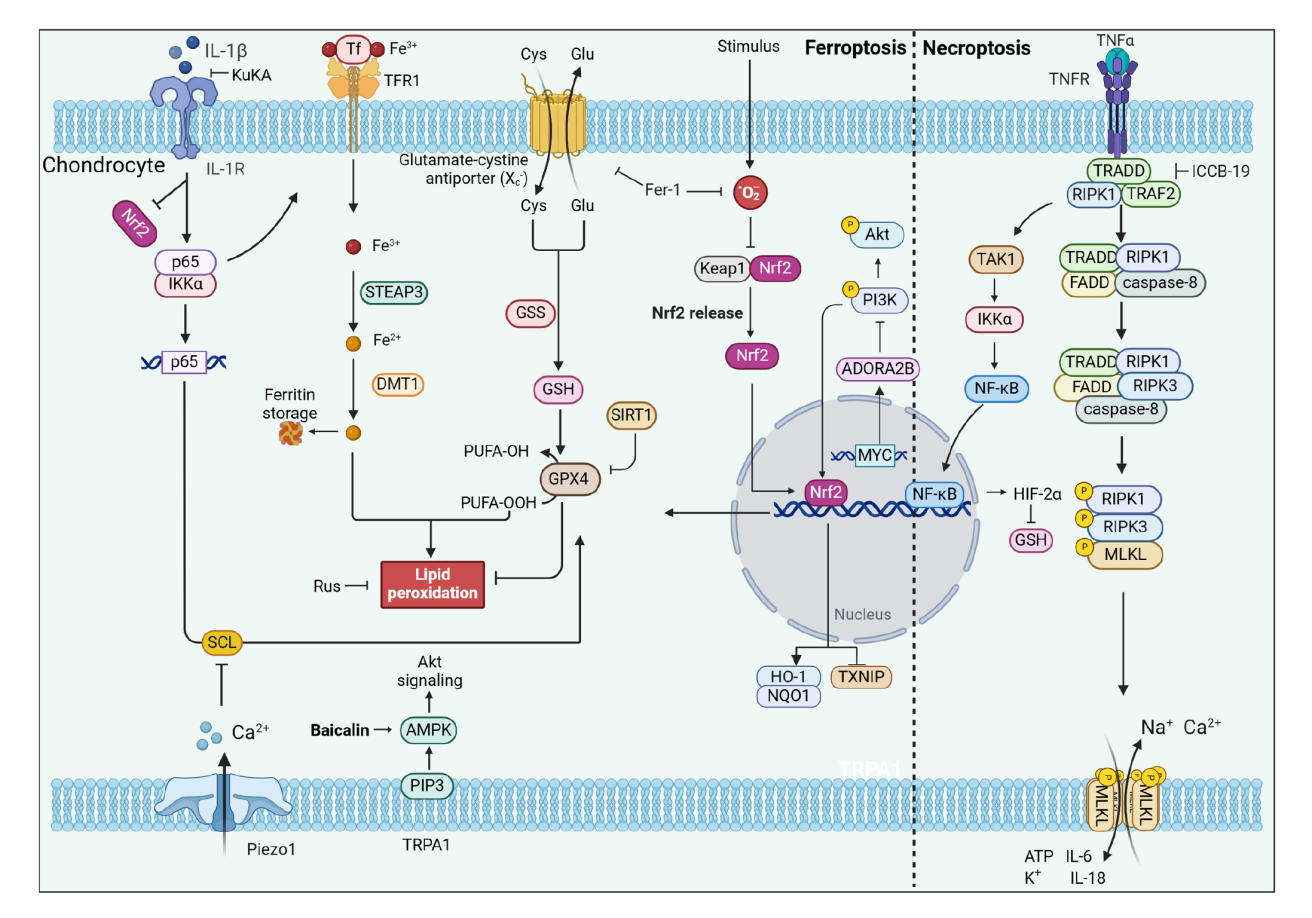
Figure 4 The metabolic pathways of chondrocyte ferroptosis and necroptosis are involved in the onset of pain in OA and the interplay between response components and those involved in other modes of cell death The figure mainly depicts the core network involved in the regulation of chondrocyte ferroptosis and the necroptosis response that causes pain in OA, which can be roughly divided into two parts. The left half is ferroptosis, and the right half is necroptosis. Kukoamine reverses several IL-1β-induced ferroptosis-related adverse effects. Brevilin A inhibits inflammatory responses and ferroptosis by modulating the SIRT1/Nrf2/GPX4 signaling. ROS further affect GPX4 metabolism through the Nrf2/NF-κB pathway. Mechanical overload promotes ferroptosis through Piezo1 activation and subsequent OA chondrocyte endocytosis of Ca2+. These mechanisms exhibit cooperative functions, leading to cellular ferroptosis. The left half is the necroptosis pathway. TRADD is a key upstream molecule for TNF-α signaling, and in TRADD deficiency, TNF-α-associated OA-like phenotypes, including inflammatory responses, extracellular matrix degradation, apoptosis, and chondrocyte necroptosis, can be inhibited by suppressing the activation of RIPK1/TAK1/NF-κB signaling and restoring impaired autophagy. ICCB-19 is a selective inhibitor of TRADD. KuKA: Kukoamine.

Other studies have reported that the RIP1/bone morphogenetic protein 7 axis induces necroptosis in chondrocytes, a unique form of cell death regulated by bone morphogenetic protein 7, a recently discovered target of RIP1 [21]. Tumor necrosis factor receptor-associated death domain protein (TRADD), which belongs to the TNF-α signaling pathway, can inhibit TNF-α-induced OA-like symptoms in mice lacking TRADD. This inhibition prevents RIPK1/TAK1/NF-κB signaling activation and restores impaired autophagy, which inhibits the inflammatory response, extracellular matrix degradation, apoptosis, and chondrocyte necroptosis. ICCB-19, a specific TRADD blocker, also inhibits chondrocyte necroptosis [21] ( Figure 4).

## Chinese and Western Medicine for the Treatment of Pain in OA Patients

Activation of the adenosine A3 receptor can slow OA progression and relieve pain by inhibiting ROS/NLRP3/GSDMD signaling [108]. Green-lipped mussels reduce necroptosis and alleviate OA pain and cartilage degeneration by suppressing the expression levels of the necroptosis mediators RIPK1, RIPK3, and MLKL in OA chondrocytes [22]. Studies have indicated that AZ-628 can decrease inflammation and cell death in cartilage cells by blocking specific cellular pathways, ultimately reducing cell death and pain in OA [109]. Icariin prevents pyroptosis and cell death in OA by inhibiting the NLRP3 signaling-mediated caspase-1 pathway and ferroptosis by downregulating TFR1 expression, activating the Xc ^-^/GPX4 axis, and inhibiting ferroptosis, thereby relieving pain symptoms [ 88, 110]. Morroniside blocks OA chondrocyte pyroptosis by inhibiting NF-κB activity [111]. Similarly, licorice chalcone A negatively suppresses pyroptosis in chondrocytes with OA by blocking the NLRP3 inflammasome [11]. USPs, such as USP7, are categorized as deubiquitinating enzymes within the ubiquitin-specific protease group. The inhibition of USP7 mitigates H _2_O _2_-induced chondrocyte injury and pyroptosis by disrupting the NOX4/NLRP3 signaling pathway [97]. Ruscogenin delays the progression of OA by preventing chondrocyte death via modulation of the Nrf2/SLC7A11/GPX4 signaling pathway [99]. Levo-tetrahydropalmatine may reduce neuroinflammation by inhibiting the Clec7a/MAPK/NF-κB/NLRP3 axis, thereby alleviating neuropathic pain [112]. Maresin1 inhibits NLRP3-induced cellular pyroptosis by inhibiting NF-κB signaling, which decreases inflammatory reactions and relieves pain [113]. Table 3 describes relevant means of targeting novel types of cell death for the treatment of pain in OA.

**Table:** **Table 3** Relevant means of targeting novel cell death for the treatment of pain in OA

Treatment	Cell	Model	Mechanism	Result	Ref.
Baihu-Guizhi decoction	SMs, FLS	Adjuvant induced Arthritis model	TLR-4/c-Fos/IL-2/TNF-α, NLRP3	Inhibition of TLR-4-mediated activation of NLRP3 inflammatory vesicle signaling inhibits SM and FLS pyroptosis, restores immunomodulatory and anti-inflammatory activity, increases pain threshold	[93]
Morroniside	Chondrocytes	Destabilization of medial meniscus model	NF-κB, NLRP3/caspase-1	Prevents cartilage matrix degradation and reduces chondrocyte pyroptosis and apoptosis	[111]
Licorice chalcone A	Chondrocytes	Destabilization of medial meniscus model	NLRP3	Inhibition of chondrocyte pyroptosis	[11]
USP7 inhibitor P22077	Chondrocytes	Monosodium iodoacetate model	NOX4/NLRP3	Irreversibly inhibits USP7 activity, suppresses USP7 mRNA and protein expression, promotes NLRP3 ubiquitination levels, blocks activation of inflammatory vesicles, and inhibits chondrocyte pyroptosis	[97]
A3 receptor sitimulator	Chondrocytes	Anterior cruciate ligament transection model	ROS/NLRP3/GSDMD	Activation of the adenosine A3 receptor inhibits ROS-initiated NLRP3 inflammasome activation and chondrocyte pyroptosis	[108]
Electro-acupuncture	Chondrocytes	Monosodium iodoacetate-induced model	NLRP3	Inhibition of NLRP3 inflammatory vesicle and pyroptosis-associated protein production reduces pyroptosis and attenuates mechanically abnormal pain and joint swelling	[114]
Ruscogenin	Chondrocytes	Destabilization of medial meniscus model	Nrf2/SLC7A11/GPX4	Inhibits chondrocytes ferroptosis	[99]
Green-lipped mussel	Chondrocytes	Monosodium iodoacetate-induced model	RIPK1/RIPK3/MLKL	Reduction of NF-κB expression, inhibition of pro-inflammatory cytokines and mediators of necroptosis, and inhibition of chondrocyte necroptosis and inflammation	[22]
AZ-628	Chondrocytes, osteoclast	Destabilization of medial meniscus model	MAPK, NF-κB, RIP3	Inhibits phosphorylation of RIP3 protein downstream of RIP1, thereby inhibiting necroptosis of chondrocytes; inhibits RANKL-induced degradation of IκBα as well as phosphorylation of P65 and IκBα, thereby decreasing osteoclast formation; reverses chondrocyte catabolism	[109]
Icariin	Synovial cells	LPS-induced model	Xc ^–^/GPX4	Inhibition of synoviocyte ferroptosis, reduction of MDA and iron content, and reduction of ferroptosis-related protein expression	[88]

## Outlook

While conservative and surgical treatments exist for OA-related pain conditions, their effectiveness is limited. This underscores the urgent need for new therapeutic approaches and personalized treatment plans that directly target the causes of pain. Some complementary treatments can serve as an addition to traditional therapies for OA. However, their efficacy and safety need to be clarified. For example, the American College of Rheumatology recommends the use of ultrasound and acupuncture, whereas the Osteoarthritis Research Society International advises against laser therapy, acupuncture, and percutaneous electrical stimulation. Various therapeutic approaches for OA have several limitations, and the underlying etiologic mechanisms are not well understood.

Our review reveals that targeting novel cell death pathways to multiple functional cells in OA is beneficial for curbing adverse phenotypes and alleviating OA pain. Further investigations are needed to determine whether the diverse novel modes of cell death undergo transformation or interact with each other. Targeting these novel modes of cell death may hold promise as a therapeutic approach for managing pain associated with various bone-related conditions. Will the combined inhibition of these types of cell death offer better control of pain in patients with bone-related diseases? Or does such a combination decrease the therapeutic effect of each novel form of cell death on pain?
